# Impact of cessation of regular cataract surgery during the COVID pandemic on the rates of posterior capsular rupture and post-operative cystoid macular oedema

**DOI:** 10.1038/s41433-022-01958-y

**Published:** 2022-02-03

**Authors:** Korina Theodoraki, Khayam Naderi, Chun Fung Jeffrey Lam, Jit Kai Tan, Ashmal Jameel, Lily Lai, Luis Onrubia Garcia, Sancy Low, Mani Bhogal, Scott Robbie, David O’Brart

**Affiliations:** 1 Department of Ophthalmology, St. Thomas’ Hospital, Lambeth Palace Road, London, SE1 7EH UK; 2 King’s College, London, WC2R 2LS UK

**Keywords:** Outcomes research, Health services

## Abstract

**Background/objectives:**

During the COVID-19, elective cataract surgery (CS) was significantly curtailed. We investigated whether consequent reduction of micro-surgical skills practice might lead to higher operative complications.

**Methods:**

Single-centre, electronic note review of consecutive patients undergoing CS during three periods: 1^st^ February 2019 to 13^th^ January 2020 (P1) prior to pandemic; 3rd June 2020 to 11th January 2021 after 1^st^ lockdown (P2); and 25^th^ January to 30^th^ July 2021 (P3) after/during second lockdown.

**Results:**

2276 operations occurred during P1, 999 during P2, 846 during P3. During P1, posterior capsular rupture (PCR) rate was 1.67%, similar to P2 (1.30%, *p* = 0.54), but lower than P3 (3.55%, *p* = 0.002). There was no difference in PCR risk percentage scores between routine and PCR cases during P1 (1.90% vs 2.03%, *p* = 0.83), P2 (2% vs 2.18%, *p* = 0.18), or P3 (1.87% vs. 2.71%, *p* = 0.08). During P2 and P3, there was a higher rate of cystoid macular oedema (CMO) compared with P1 (4.9% and 6.86% vs. 1.93%, *p* = 0.0001), with no differences in proportion of diabetics or cases with CMO in combination with PCR. There was no difference in surgeons grade experiencing PCR.

**Conclusions:**

In P3 following 9 months of curtailed elective CS, PCR rates were increased across all surgeon grades, occurring in cases with similar risk percentage scores. CMO rates were increased during COVID and not related to proportion of diabetics or increased PCR rates. The reduction in elective CS during the pandemic was associated with more complications, perhaps due to attenuation of microsurgical skills.

## Introduction

Cataracts are a major cause of blindness. With an aging population, population growth, and mounting rates of age-related diseases associated with cataract, the demand for cataract surgery (CS) is increasing [1]. CS is the most common surgery undertaken for pathology with > 4 million procedures performed in the European Union in 2016 [2] and 452,000 in the UK during 2018-19 [3]. With modern, microscopic surgical techniques and intraocular lenses with advanced optics, visual and refractive outcomes are excellent with 95% of healthy eyes achieving 0.3 LogMAR corrected acuity or better [3]. Indeed, CS is one of the commonest and successful interventions in medicine, with high patient and surgeon outcome expectations [4].

On 11th March 2020, The World Health Organization declared the coronavirus (COVID-19) outbreak a global pandemic [5]. In spring 2020, the United Kingdom (UK) went into lockdown measures on 23rd March 2020 [6]. As part of these measures, all non-essential medical appointments were cancelled, and elective surgery suspended. As such, routine CS ceased for 2 months and was not recommenced in most National Health Units (NHS) units until June 2020. Such measures impacted service provision, generating a surgical backlog [7]. Patients that had been pre-assessed, consented and placed on the waiting list were cancelled and delayed. In a survey during the first lockdown of patients on our CS waiting list, 65% felt that their quality of life (QOL) had deteriorated due to worsening eyesight due to delayed surgery [8]. This reported worsening of vision in patients already assessed, coupled with the knowledge that there were likely to be substantial numbers of individuals in the community with visually significant cataract who were remaining undiagnosed because of lockdown measures, led to a perception that following resumption of elective surgery more advanced cataract cases, with greater surgical challenges, might be encountered.

In addition to this backlog, many ophthalmic surgeons, some for the first time, ceased undertaking micro-surgery with all its associated complexities and skill sets for several months, being redeployed to medical and critical care units or remaining within ophthalmic departments to provide emergency out-patient care. Breaks from performing regular surgery have been shown to lead to deterioration in technical skills [9]. From observational studies of medical practitioners, beyond 6 months, de-skilling, particularly of fine-motor skills, is rapid and followed by a slower skill degradation with time [10–12], with the rates of deskilling varying between individuals, due to mitigating factors such as stress, anxiety, and lost confidence [10–12]. Reduced surgical performance can compromise patient safety, especially with the possible presentation of more complex cases. This might be perceived as important in relation to CS, given the frequency at which it is undertaken, the high micro-surgical skills that modern CS demands, and high patient and surgeon expectations [2, 4, 5].

The aim of this study was to investigate these issues by examining the electronic medical records (EMR) of all patients in our unit undergoing elective CS, with reference to surgical complications and case complexity, during two approximate 7-month periods following the first lockdown period in 2020 and during and after the second lockdown period in the UK in 2021 and comparing them to an 11-month period prior to the pandemic.

## Subjects and methods

This study was conducted at a public university hospital in the UK. It was approved by our institution’s audit and quality improvement project team (audit number 11822). Data collection adhered to the Tenets of the Declaration of Helsinki and UK Data Protection Act.

Patient data were collected from our EMR system (OpenEyes™, Sunderland, Tyne and Wear, England). As well as providing patient demographics, past medical history, details of consultations and peri-operative data, the software calculates a posterior capsular rupture (PCR) percentage risk rate where clinical characteristics, identified from the National (UK) Ophthalmology Database as risk factors for PCR [13], such as cataract density, axial length, ocular and systemic co-morbidities, are input in patients’ records.

All patients who underwent CS within our unit, performed between February 2019 and July 2021 were included. Patients that had cataract surgery combined with glaucoma, corneal or vitreoretinal surgery were excluded. Reference was made to demographic data, surgeon’s seniority, surgical complexity based on the PCR risk score and peri- and post-operative complications. The different time points examined were 1st February 2019 to 13th January 2020 (P 1) (prior to the COVID-19 pandemic), from 3rd June 2020 to 11th January 2021 after 1^st^ lockdown (P 2), and 25th January 2021 to 30th July 2021 (P 3) after and during second lockdown. Documented findings collected included patient age, gender, right or left eye, pre-cataract surgery best-corrected distance visual acuity (BCDVA), final postoperative BCVA, cystoid macular oedema (CMO) rates, and diabetic status. Visual acuities were recorded in LogMAR acuity. Visual acuities of counting fingers, hand movements, light perception, and no perception of light were rounded up to 1.8 (LogMAR), 2.3, 2.8, and 3.0, respectively [14].

Statistical analysis was performed using GraphPad Prism version 8.0.0 for Mac (GraphPad Software, San Diego, California USA). Data sets was assessed for normality using the Shapiro-Wilk test. For parametric data unpaired T-test was used to compare means between groups. For non-parametric data sets, the Mann-Whitney was used to compare the medians between different groups, and the Wilcoxon test to compare medians between the same groups. Fisher’s exact tests were used to compare the proportion of cases between groups.

## Results

2276 cataract surgeries were performed during P1, 999 surgeries in P2, and 846 in P3 (Table 1). The cohort was similar in terms of demographics (age, gender, laterality). Patients during P3 had statistically significant worse BCDVA compared to P1 (*P* < 0.0001) (Table 1).

**Table 1:** Patient Demographics.

	P1	P2	P1 vs. P2 *P* value	P3	P1 vs. P2 *P* value
Age (years)	71 (17–96)	71 (14–96)	*P* = 0.079	70 (31–93)	*P* = 0.024
Gender (Male:Female)	1036:1250	431:568	*P* = 0.32	364:482	*P* = 0.31
Eye (Right:Left)	1141:1135	523:476	*P* = 0.25	412:434	*P* = 0.49
Pre-operative BCDVA (logMAR)	0.30 (−0.20 to 3.00)	0.30 (−0.20 to 2.80)	*P* = 0.47	0.40 (−0.10 to 2.80)	*P* < 0.0001

*P1* Period 1, *P2* Period 2, *P3* Period 3, *BCDVA* Test-corrected distance visual acuity.

Fisher’s Exact Test used to compare differences between two groups for gender and eyes. Statistical significance set at *p* < 0.05. Mann-Whitney Test used to compare differences between groups for age and BCDVA.

There were 38 cases of PCR in P1 (1.67%), 13 in P2 (1.30%), and 30 in P3 (3.55%) (Table 2). There was a significant increase in the PCR rate when P1 and P3 (*p* = 0.0022) and when P2 and P3 were compared (*p* = 0.0017). In terms of PCR with vitreous loss (PCRVL) there were 28 cases in P1 (1.23%), 7 in P2 (0.7%), and 27 in P3 (3.19%). There was an increase in the PCRVL rate when P1 and P3 (*p* = 0.006) and when P2 and P3 were compared (*p* = 0.001) Table 2).

**Table 2:** Vitreous Loss and Post-operative Cystoid Macular Oedema Rates.

	P1	P2	P1 vs. P2 *p*-value	P3	P1 vs. P3 *p*-value	P1 vs. P2 + P3 *p*-value	P2 vs. P3 *p*-value
Intra-operative PCR Rate (%)	38/2276 (1.67%)	13/999 (1.30%)	*P* = 0.54	30/846 (3.55%)	*P* = 0.002	*P* = 0.14	*P* = 0.001
Intra-operative PCRVL Rate (%)	28/2276 (1.23%)	7/999 (0.70%)	*P* = 0.20	27/846 (3.19%)	*P* = 0.0006	*P* = 0.12	*P* < 0.0001
Post-operative CMO Rate (%)	44/2276 (1.93%)	49/999 (4.9%)	*P* < 0.0001	58/846 (6.86%)	*P* < 0.0001	*P* < 0.0001	*P* = 0.089
Median waiting times (days) from listing to surgery in PCR cases	41.5 (1–118)	30 (6–301)	*P* = 0.99	53 (4–224)	*P* = 0.29	*P* = 0.43	*P* = 0.55
Post-operative CMO cases with diabetes (%)	20/44 (45.45%)	16/49 (32.65%)	*P* = 0.29	23/58 (39.66%)	*P* = 0.69	*P* = 0.36	*P* = 0.55
Post-operative CMO cases with PCR (%)	4/44 (9.09%)	0/49 (0%)	*P* = 0.047	1/58 (1.72%)	*P* = 0.16	*P* = 0.026	*P* > 0.99

*P1* Period 1, *P2* Period 2, *P3* Period 3, *SD* Standard Deviation, *PCR* All cases of Posterior Capsular Rupture with and without vitreous loss), *PCRVL* Posterior Capsular Rupture with Vitreous Loss, *CMO* Cystoid Macula Oedema.

Fisher’s Exact Test used to calculate differences between two groups. Statistical significance set at *p* < 0.05.

During P1, median PCR percentage risk score was 1.90% (0.30–53.34) in routine and 2.03% (0.84–52.26) in PCR cases (0.83). The median risk score during P2 was 2% (0.27–37.12) in routine and 2.18% (1.36–10.97) in PCR cases (*p* = 0.18). In P3 the median risk score was 1.87% (0.27–33.80) in routine and 2.71% (0.42–7.89) in PCR cases (*p* = 0.08) (Table 3). There was no significant difference in the median risk scores in cases experiencing PCR in P1 compared to P2 (*p* = 0.28) and P3 (*p* = 0.32) (Table 3).

**Table 3:** Intra-operative Posterior Capsular Rupture Percentage Risk Scores: Routine Cases versus Vitreous Loss Cases.

	P1 R Cases Median (Range)	P1 PCR Cases Median (Range)	P1 R vs. PCR Cases *p*-value	P2 R Cases Median (Range)	P2 PCR Cases Median (Range)	P2 R vs. PCR Cases *p*-value	P3 R Cases Median (Range)	P3 PCR Cases Median (Range)	P3 R vs. PCR Cases *p*-value
Intra-operative PCR Risk (%)	1.90% (0.30–53.34)	2.03% (0.84–52.26)	*P* = 0.83	2.00% (0.27–37.12)	2.18% (1.36–10.97)	*P* = 0.18	1.87% (0.27–33.80)	2.71% (0.42–7.89)	*P* = 0.08

*P1* Period 1, *P2* Period 2, *P3* Period 3, *SD* Standard Deviation, *PCR* All cases of Posterior Capsular Rupture with and without vitreous loss, *R* Routine, *VL* Vitreous Loss.

Fisher’s Exact Test used to calculate differences between two groups. Statistical significance set at *p* < 0.05.

Mann-Whitney Test used to compare medians between two groups. Statistical significance set at *p* < 0.05.

There were 44 cases of post-operative CMO in P1 (1.93%), 49 in P2 (4.9%) and 58 in P3 (6.86%) (Table 2). There was a statistically significant (*p* = 0.0001) increase in the CMO rate when P1 is compared with P2 (*p* < 0.0001), P3 (*p* < 0.0001) and P2 + P3 (*p* < 0.001). Amongst patients who developed CMO, the proportion of diabetics was similar between P1 and P2 (45.45% vs. 32.65%, *p* = 0.29), and P1 and P3 (45.45% vs. 39.66%, *p* = 0.69) (Table 2). There were no differences in numbers of cases with combined PCR and CMO between P1 (9.09%) versus P3 (1.72%) (*p* = 0.16), although rates of combined PCR and CMO were significantly higher in P1 compared with P2 (0%) (*p* = 0.047) and P2 and P3 combined (*p* = 0.026). There were no differences in pre-operative BCDVA measurements between eyes that experienced CMO and eyes that did not for P1 (0.3 LogMAR (0.0–2.8) vs. 0.3 LogMAR (−0.2 to 3.00) *p* = 0.62), P2 (0.36 LogMAR (0.02.8) vs. 0.3 LogMAR (−0.2 to 3.0) *p* = 0.36) and P3 (0.41 LogMar (0.0–2.8) vs. 0.4 LogMAR (−0.1 to 2.8) *p* = 0.76).

There was no significant difference in terms of seniority of surgeons who experienced PCR (Table 4), with no difference in the proportion of PCR cases operated on by consultant surgeons during P1 compared to P2 (*p* = 0.30) and P3 (*p* = 0.24) (Table 4).

**Table 4:** Intra-operative Percentage of Operations performed by Consultant Surgeons in Cases with Posterior Capsular Rupture during Period 1 compared to Periods 2 and 3.

	P1 PCR Cases Median (Range)	P2 PCR Cases Median (Range)	P1 PCR vs. P2 PCR Cases *p*-value	P3 PCR Cases Median (Range)	P1 PCR vs. P3 PCR Cases *p*-value	P1 PCR vs. P2 + P3 PCR Cases *p*-value
Consultant: Non-Consultant	10:28	6:7	*P* = 0.30	11:19	*P* = 0.43	*P* = 0.24

*P1* Period 1, *P2* Period 2, *P3* Period 3, *SD* Standard Deviation, *PCR* All cases of Posterior Capsular Rupture with and without, VL Vitreous Loss.

Fisher’s Exact Test used to compare differences between groups. Statistical significance set at *p* < 0.05.

Where data was available, BDCVA significantly improved for all time periods in both routine and cases with PCR during all three periods (Table 5). There was no significant difference in pre-operative BCDVA in cases that experienced PCR in P2 (1.48 LogMAR (0.0–2.8)) and P3 (0.78 LogMAR (0.0–2.8)) compared with P1 (0.3 LogMAR (0.0–2.8)) (P1 vs. P2 *p* = 0.1 and P1 vs. P3 *p* = 0.16) (Table 5).

**Table 5:** Pre-operative versus Post-operative Best-Corrected Distance Visual Acuity.

	Pre-operative R Cases Median (Range)	Post-operative R Cases Median (Range)	*p*-value (R cases pre-operative BCVA vs post-operative BCVA)	Pre-operative PCR Cases Median (Range)	Post-operative PCR Cases Median (Range)	*p*-value (PCR cases pre-operative BCVA vs post-operative BCVA)
P1 BCVA (logMAR)	0.30 (−0.20 to 3.00)	0.10 (−0.24 to 2.80)	*P* < 0.0001 (*N* = 1721)	0.30 (0.00–2.80)	0.10 (−0.10 to 2.30)	*P* = 0.0054 (*N* = 30)
P2 BCVA (logMAR)	0.30 (−0.20 to 2.80)	0.080 (−0.20 to 2.80)	*P* < 0.0001 (*N* = 920)	1.48 (0.00–2.80)	0.10 (0.00–0.70)	*P* = 0.0010
P3 BCVA (logMAR)	0.40 (−0.10 to 2.80)	0.1 (−0.10 to 2.80)	*P* < 0.0001 (*N* = 704)	0.78 (0.00–2.80)	0.20 (−0.06 to 2.30)	*P* = 0.0020 (*N* = 25)

*P1* Period 1, *P2* Period 2, *P3* Period 3, *SD* Standard Deviation, *PCR* All cases of Posterior Capsular Rupture with and without Vitreous Loss, *R* Routine, *BCVA* Best-corrected Distance Visual Acuity.

Fisher’s Exact Test used to calculate differences between two groups. Statistical significance set at *p* < 0.05.

Wilcoxon Test used to compare medians between same two groups. Statistical significance set at *p* < 0.05.

## Discussion

In the UK, during the national COVID-19 lockdown periods, elective CS surgical ceased. In addition, large numbers of ophthalmic staff were redeployed, and it would have been challenging to deliver safe standards of elective care. Following the lockdowns, when elective activity resumed, to safeguard patients and health care professionals, several measures were introduced, including social distancing within clinical settings, asking patients to self-isolate at home and have proof of a negative COVID polymerase chain reaction 3 days prior to surgery. As such, the number of patients that could be operated per list was limited and our case numbers were reduced (Table 1).

Contemporary CS with its advanced microsurgical instrumentation and techniques requires high levels of training and micro-surgical skill attainment. Many surgeons ceased undertaking routine micro-surgery with its complex skill sets for several months, while others in training had no chance to improve their skills. Breaks from performing surgery can lead to technical skills deterioration [9], with studies showing that after 6 months, rapid de-skilling of fine-motor skills occurs [10–12]. It may be postulated that such attenuation of micro-surgical might result in increased operative and post-operative complications. Such factors could be exacerbated by the presentation of more advanced cases with more mature cataracts, because of increased waiting times for surgery. For these reasons we undertook this current study, to investigate whether CS complication rates had increased and factors that might be responsible.

Our results indicated that during P3, following, and during the second lockdown, rates of PCR more than doubled and PCRVL almost trebled in our unit compared to pre-pandemic levels in P1 and those after the first lockdown P2 (Table 2). This was despite no increase in the PCR risk percentage scores (Table 3) or the proportion of PCR cases operated on by consultant surgeons. Such findings support the hypothesis that prolonged interruptions in regular CS may have a detrimental effect on micro-surgical skills resulting in increasing operative complications, and that surgeon experience is not a mitigating factor. In a published national survey, Maubon et al, found that > 50% of Ophthalmic surgeons suffer transient anxiety when returning to surgery after a hiatus as short as 8 weeks [15], with perceived operating difficulties in 30%, perceived increased surgical time and reduced surgical confidence. Our findings are supported by a study of 15689 CS operations in an NHS tertiary referral centre (Moorfields Eye Hospital), where PCR rate increased from 0.99% to 1.62% post-pandemic [16]. The authors suggested that restrictions in surgical activity during lockdown probably resulted in an increased incidence of PCR [16]. Interestingly, their documentation of increased PCR rates occurred after the first lockdown, equivalent to P2 in our study. We found no differences in PCR and PCRVL rates before (P1) and after the first lockdown (P2), with rates only increasing in P3 (Table 2). At this stage elective CS had been curtailed for almost 9 months, when fine motor skill degradation has been shown to occur [10–12]. It would be interesting to know if the PCR rates at Moorfields have showed a further increase during time periods commensurate to P3.

After and during the second lockdown (P3), when PCR and PCRVL rates significantly increased (Table 2), patients had been waiting in some cases for almost 12 months for their surgery and one would expect their cataracts have been maturing, albeit slowly. This appears to be reflected by poorer pre-operative BCDVA measurements during P3 compared to P1 (0.40 logMR vs. 0.30 logMAR, *P* = 0.0001) (Table 5), although it must be noted that this is only a surrogate maker for cataract density and can be of course influence by other factors. However, the possibility of denser cataracts presenting for surgery should be considered as a mitigating factor especially in conjunction with probable micro-surgical skills degradation. Pre-operative BCDVA is not reflected in our PCR percentage risk score calculations [13] although the presence of brunescent/ white cataracts are.

During P2 and P3, there was an increased rate of CMO compared to P1 (Table 2). In the cases with CMO, there was no difference in the proportion of diabetics across both P2 and P3 or pre-operative BCDVA or of combined cases of PCR with subsequent CMO (Table 2), suggesting that these were not important factors in increased rates. The incidence of reported post-operative CMO after CS ranges from 0.02–7.6% [17–19], dependent on the population examined, risk factors and intraoperative complications. A self-reported survey in a predominantly Caucasian population in Scotland found an incidence of 0.02% [17] while in an ethnically diverse London population with high number of diabetic patients the rate of CMO was 7.6% [18]. In our patients there was an increased incidence of CMO (*p* < 0.0001) during COVID with 49 episodes (4.9%) in P2 and 58 (6.85%) during P3. This rise is likely to be multifactorial. It could be postulated that the longer waiting times for surgery in P2 and P3 may have led to more mature cataracts presenting on the day of surgery. One of the limitations of our study is that patients waiting for surgery because of lockdown restrictions were not recalled for re-examination prior to surgery and that PCR risk percentage scores are not routinely re-calculated on the day of surgery even in patients who have had an extended waiting time for CS. Given our currently high PCR and PCRVL rates this may be something that needs to be reconsidered. Potentially denser cataracts may result in the need for higher phacoemulsification cumulative dissipated energy (CDE) and operations taking longer than usual, resulting in increased post-operative inflammation. It has been postulated increased post-operative inflammation is a risk factor for CMO [20]. Unfortunately, the operative CS time or CDE scores we not routinely recorded on our EMR, which considering the current study we may enforce. Importantly, there did not appear to be differences in pre-operative BCDVA measurements between eyes that experienced CMO and eyes that did not during any of the periods. This suggests that cataract density may not have been an important factor for CMO development in our patients, as any reduction of BCDVA associated with increased cataract density with time, would have been assumed to occur roughly equally in all eyes. However, factors such as surgical (iris) trauma and prolonged CS time due to micro-surgical skills degradation or other unidentified factors, or operative complications not picked up from our electronic data base cannot be ruled out.

There was no difference in the proportions of consultant surgeons operating on cases that experienced PCR in P1, compared to P2 and P3 (Table 4). Whilst it might be postulated that those in training with limited microsurgical experience may encounter the greatest problems when returning to surgery after a hiatus, this does not appear to be the case, with all grades experiencing problems. Our results are supported by the study from Moorfields, where consultants were found to have statistically significant increase in their PCR rates post-pandemic [16]. In our unit the lowest grade of surgeon performing CS is a Specialist Registrar Year 3 and would have had at least two years of experience. In addition, based on our percentage risk score [13], cases are stratified and those will lower scores are generally allocated to doctors in training. Such factors would affect outcomes in this current study.

BCDVA appeared to be significantly improved in all eyes following surgery and in all periods in eyes both with and without PCR (Table 5). This highlights the fact that although far from ideal, when PCR is managed carefully and correctly most patients continue to do well and have improved vision following CS.

Covid 19 has had a significant impact on ophthalmology, and we continue to adjust our services in response to the evolving nature of the pandemic. Our findings of increased PCR and PCRVL rates in the presence of similar PCR percentage risk scores in P3, suggest that a hiatus of 9 months from regular routine high volume elective CS, perhaps due to micro-surgical skills degradation, is responsible for such operative complications. How issues such as the length of time away from undertaking micro-surgery, personal circumstances, and prior surgical experience can impact ophthalmologists’ performances on returning to CS and subsequent outcomes and complications is unknown and there is little research in this area [21]. General guidelines exist [22–26], but they lack specificity or compulsion, and certainly no guidelines or criteria for returning to perform routine CS either nationally from the NHS/Royal College of Ophthalmologists or locally were put in place during the pandemic. This contrasts with equivalent ‘high reliability’ professions such as aviation. Unlike ophthalmologists and surgeons in general, commercial pilots are subject to mandatory checklists, and mental and physical screening before re-commencing flying. A minimum of three take off and landings within 90 days are an absolute requirement to be allowed to continue duties, which was altered to 1 in 60 days during the COVID-19 pandemic due to possible skills fade associated with the disruption of commercial flights [27]. Commercial pilots are also required to undertake time on flight simulators, as well as supervised flights, after any extended breaks [27, 28]. Considering the results of this current and similar recent studies [17] and with the knowledge that extensive breaks from surgery lead to skill fade [9–12], it would seem very sensible that Ophthalmology attempts to learn lessons from the aviation industry by investigating factors that may optimise returns after surgical breaks. Clearly, there is a need, to improving patient safety, of more support for surgeons of all grades when they return to surgery after an extended hiatus, with the development of robust guidelines and including perhaps mandatory time spend on surgical simulators.

### Summary

#### What is known about this topic

- Breaks from performing surgery lead to deterioration in technical skills, especially fine motor skills after 6 months.
- During the COVID-19 pandemic lockdown periods all elective surgery, including cataract surgery which necessitates fine motor skills, was cancelled.

#### What this study adds

- After 9 months of curtailed elective cataract surgery rates of posterior capsular rupture increased across all surgeon grades and was not related to case complexity.
- Post-operative cystoid macula oedema rates increased during COVID and were not related to the proportion of diabetics or increased PCR rates.
- The reduction in elective cataract surgery during the pandemic was associated with more complications, perhaps due to attenuation of microsurgical skills.
- There appears to be a need for more support and guidelines for ophthalmic surgeons of all grades when they return to surgery after an extended hiatus, to improve patient safety.

## References

[1] Minassian D, Reidy A. Future Sight Loss. UK 2. https://www.rnib.org.uk/…/general-research/future-sight-loss-uk-2.

[2] Surgical operations and procedures statistics. https://ec.europa.eu/eurostat/statistics-explained/index.php?title=Surgical_operations_and_procedures_statistics.

[3] Day AC, Donachie PHJ, Sparrow JM, Johnston RL, Royal College of Ophthalmologists’ National Ophthalmology Database. (2015). The Royal College of Ophthalmologists’ National Ophthalmology Database study of cataract surgery: Report 1, visual outcomes and complications. Eye Lond Engl.

[4] Pager CK (2004). Expectations and outcomes in cataract surgery: A prospective test of 2 models of satisfaction. Arch Ophthalmol Chic Ill 1960.

[5] Organization, WH *WHO Director-General’s opening remarks at the media briefing on COVID-19-11**March 2020*. (Geneva, Switzerland, 2020).

[6] Timeline of UK coronavirus lockdowns, March 2020 to March 2021. https://www.instituteforgovernment.org.uk/sites/default/files/timeline-lockdown-web.pdf.

[7] Aggarwal S, Jain P, Jain A (2020). COVID-19 and cataract surgery backlog in Medicare beneficiaries. J Cataract Refract Surg..

[8] Naderi K, Maubon L, Jameel A, Patel DS, Gormley J, Shah V (2020). Attitudes to cataract surgery during the COVID-19 pandemic: A patient survey. Eye Lond Engl.

[9] Skills fade literature review. https://www.gmc-uk.org/about/what-we-do-and-why/data-and-research/research-and-insight-archive/skills-fade-literature-review.

[10] Ahya SN, Barsuk JH, Cohen ER, Tuazon J, McGaghie WC, Wayne DB (2012). Clinical performance and skill retention after simulation-based education for nephrology fellows. Semin Dial.

[11] Walsh A, Gold M, Jensen P, Jedrzkiewicz M (2005). Motherhood during residency training: Challenges and strategies. Can Fam Physician Med Fam Can..

[12] Hiemstra E, Kolkman W, van de Put MAJ, Jansen FW (2009). Retention of basic laparoscopic skills after a structured training program. Gynecol Surg.

[13] Narendran N, Jaycock P, Johnston RL, Taylor H, Adams M, Tole DM (2009). The Cataract National Dataset electronic multicentre audit of 55,567 operations: Risk stratification for posterior capsule rupture and vitreous loss. Eye (Lond).

[14] Bonnan M, Valentino R, Debeugny S, Merle H, Fergé JL, Mehdaoui H (2018). Short delay to initiate plasma exchange is the strongest predictor of outcome in severe attacks of NMO spectrum disorders. J Neurol Neurosurg Psychiatry.

[15] Maubon L, Nderitu P & O’Brart. DPS Returning to cataract surgery after a hiatus: A UK survey report. Eye Lond Engl. 2021. 10.1038/s41433-021-01717-5. 10.1038/s41433-021-01717-5 PMC8343362 34363047

[16] Matarazzo F, Phylactou M, Day AC, & Maurino V. The effect of surgical abstinence on the risk of posterior capsule rupture during cataract surgery. J Cataract Refract Surg. 2021. 10.1097/j.jcrs.0000000000000741. 10.1097/j.jcrs.0000000000000741 34261984

[17] Erikitola O-O, Siempis T, Foot B, Lockington D (2021). The incidence and management of persistent cystoid macular Oedema following uncomplicated cataract surgery-a Scottish Ophthalmological Surveillance Unit study. Eye Lond Engl.

[18] Oyewole K, Tsogkas F, Westcott M, Patra S (2017). Benchmarking cataract surgery outcomes in an ethnically diverse and diabetic population: final post-operative visual acuity and rates of post-operative cystoid macular oedema. Eye.

[19] Ching H-Y, Wong AC, Wong C-C, Woo DC, Chan CW (2006). Cystoid macular oedema and changes in retinal thickness after phacoemulsification with optical coherence tomography. Eye Lond Engl.

[20] De Maria M, Coassin M, Iannetta D, Fontana L (2021). Laser flare and cell photometry to measure inflammation after cataract surgery: A tool to predict the risk of cystoid macular edema. Int Ophthalmol.

[21] Ho DK, Chiu AKC, Tung SW. Restarting cataract surgery after an extended period out of training: a perspective from the United Kingdom. Eye. 2020. 10.1038/s41433-020-01224-z. 10.1038/s41433-020-01224-z PMC8182803 33067548

[22] Royal College of Ophthalmologists. Mitigating the impact of COVID-19 on ophthalmology training. 2020. https://www.rcophth.ac.uk/wp-content/uploads/2020/11/Mitigating-the-effects-of-COVID-19-on-ophthalmologists-in-training-November-2020.pdf.

[23] Royal College of Anaesthetists. Returning to work after a period of absence. London: RcoA; 2015. https://www.rcoa.ac.uk/system/files/ReturnToWork2015.pdf.

[24] Health Education England, NHS. Supported return to training. 2017. https://www.hee.nhs.uk/sites/default/files/documents/Supported%20Return%20to%20Training.pdf.

[25] Academy of Medical Royal Colleges. Return to practice guidance: 2017 revision. London: AoMRC; 2017. https://www.aomrc.org.uk/wp-content/uploads/2017/06/Return_to_Practice_guidance_2017_Revison_0617-2.pdf.

[26] British medical Association. Returning to clinical practice after absence. 2020. https://www.bma.org.uk/advice-and-support/career-progression/applying-for-a-job/returning-to-clinical-practice-after-absence.

[27] European Union Aviation Safety Agency. Guidelines for handling exemptions to flight crew recent experience requirements in the field of commercial air transport operations. 2020. https://www.easa.europa.eu/sites/default/files/dfu/easa_guidelines-exemption_fc_recency_oro.fc_.100-fcl.060_issue_3_09.07.20.pdf.

[28] International Air Transport Association. Guidance for managing pilot training and licensing during COVID-19 operations. 2020. https://www.iata.org/contentassets/c0f61fc821dc4f62bb6441d7abedb076/iata-guidance-for-managing-pilot-training-licensing-during-covid19.pdf.

